# High-Intensity Focused Ultrasound Technology: Physical Principles, Biological Effects, and Clinical Applications in Facial and Neck Rejuvenation

**DOI:** 10.3390/biomedicines14102158

**Published:** 2026-09-24

**Authors:** Maricarmen Chacín, Ivana Vera, Pablo Duran, Valentina Gutiérrez, Amurabit Santos, Julio Cesar Contreras-Velázquez, Ana Milena Rojas Calero, Lady Giovanna Muñoz, Antony Elias Lombana-Jiménez, José Alban Londoño Arias, Carlos Hernán González Parias, Valmore Bermúdez

**Affiliations:** 1 Facultad de Ciencias de la Salud, Universidad Simón Bolívar, Barranquilla 080001, Colombia; valmore.bermudez@unisimon.edu.co; 2 Endocrine and Metabolic Diseases Research Center, School of Medicine, University of Zulia, Maracaibo 4002, Venezuelapabloduran1998@gmail.com (P.D.); amurabit21@gmail.com (A.S.); 3 Departamento de Productividad e Innovación, Universidad de la Costa, Barranquilla 080001, Colombia; 4 Facultad de Ingenierías, Institución Universitaria Antonio José Camacho, Cali 760050, Colombia; 5 Facultad de Ciencias Administrativas y Económicas, Tecnológico de Antioquia Institución Universitaria, Medellín 050034, Colombia

**Keywords:** HIFU, facial rejuvenation, focused ultrasound, aesthetic medicine

## Abstract

High-Intensity Focused Ultrasound (HIFU) has emerged as a revolutionary non-invasive technology in aesthetic medicine, enabling targeted tissue remodelling and skin rejuvenation through precise thermal and mechanical effects. This narrative review provides a comprehensive examination of the physical foundations of HIFU, including acoustic wave propagation, piezoelectric energy conversion, and transducer design. It also explores its biological mechanisms, including collagen denaturation, neocollagenesis, fibroblast activation, and extracellular matrix remodelling. Clinically, HIFU has shown significant efficacy in improving skin laxity, reducing wrinkles, and enhancing facial and neck contours, with a favourable safety profile supported by multiple studies. The review also examines the limitations of conventional HIFU devices and describes the newest emerging multi-transducer platform with multi-depth cartridges and dynamic applicators. Key gaps in the literature include the scarcity of randomised controlled trials, limited data on higher Fitzpatrick skin phototypes, and the lack of standardised treatment protocols. By synthesising current evidence, this work positions HIFU as an established modality in aesthetic dermatology. A multi-transducer HIFU platform is discussed as a technically interesting emerging platform whose proposed advantages in precision, homogeneity, and comfort remain to be confirmed by independent comparative and randomised studies.

## 1. Introduction

Ultrasound technology was first discovered in 1880 when Pierre and Jacques Curie studied the effects of mechanical vibration on quartz crystals, leading to the identification of the piezoelectric effect, which is considered the core principle of ultrasound. Ultrasound was later introduced into medicine for fetal imaging and echocardiography, becoming an essential diagnostic tool. However, it was not until 1932 that a new technique, High-Intensity Focused Ultrasound (HIFU), was proposed. This non-invasive therapeutic modality is based on the biophysical effects of ultrasound propagation to modify the cell micro- and macro-environment, enabling advances in areas such as neurosurgery, oncology, and ophthalmology [1,2].

Currently, HIFU has gained wide popularity in aesthetic dermatology as a non-invasive treatment for skin tightening and rejuvenation. Increasing temperature at a specific point produces precise thermal coagulation points at a specific depth, most commonly targeting the superficial musculoaponeurotic system (SMAS). As a result, it induces skin collagen remodelling and contraction, gradually enhancing skin firmness. This mechanism allows for a broad spectrum of applications, including facial and neck rejuvenation [3,4].

This therapeutic principle has marked a milestone in modern aesthetic medicine by providing an effective, non-surgical option for treating skin laxity and wrinkles. Based on this foundation, conventional HIFU systems, including focused and superficial HIFU, have been developed and have demonstrated positive outcomes in lifting and tightening dermal and subdermal tissues. Nevertheless, the pursuit of more effective, comfortable, and adaptable treatments has driven significant technological innovation. Among these, the multi-transducer HIFU platform system stands out as a next-generation system that integrates multi-transducer technology and novel applicators, such as the pen-type applicator. This evolution enables tissue remodelling across multiple skin layers, optimising the patient experience and broadening the therapeutic potential of focused ultrasound [5].

Despite technological advances and the growing popularity of HIFU in aesthetic dermatology, both this technique and its latest evolution, the line and pen-type applicators of this platform system, remain poorly understood by many professionals and patients, particularly regarding their physical principles, mechanisms of action, and therapeutic benefits. Within this context, this review aims to provide a comprehensive overview of HIFU performance, discussing its biological effects on tissues and its clinical impact on facial and cervical rejuvenation. In addition, we aimed to conduct a critical analysis of its aesthetic applications, compile clinical evidence to compare the efficacy of conventional HIFU with that of other advanced-technology devices, and position the latter within the evolving landscape of non-invasive focused ultrasound treatments [6,7].

## 2. Materials and Methods

This narrative review was conducted following a comprehensive search of the existing literature through July 2025. Major electronic databases, including PubMed, Embase, Scopus, Web of Science, and Google Scholar, were systematically searched, using the keywords “HIFU”, “high-intensity focused ultrasound”, “multitransducer ultrasound”, “skin tightening”, “facial rejuvenation”, “aesthetic dermatology”, “SMAS lift”, “collagenesis”, and “multi-depth ultrasound”, as well as relevant combinations of these terms. Original peer-reviewed research articles, systematic reviews, meta-analyses, and pertinent preclinical studies published in English were prioritised. Conference abstracts and data provided by manufacturers were also considered when applicable, with appropriate acknowledgement of their preliminary nature or potential for bias, to provide a comprehensive perspective on this emerging technology.

## 3. Physical Principles of HIFU

HIFU is a non-invasive therapeutic technology based on ultrasound that several research laboratories have studied over the last few years to understand its potential benefits in the medical field. To this end, each of its physical fundamentals needs to be studied, including ultrasound beam generation, the tools employed, piezoelectricity, and the penetration frequencies of HIFU waves, all of which enable the effective therapeutic use of this technology [8,9].

### 3.1. Principles of Acoustic Wave Propagation and Focusing

HIFU uses longitudinally propagating acoustic waves that must be selectively focused on tissues, traversing multiple tissue layers before reaching the target region. At each layer, part of the energy carried by the sound wave is reflected (impedance), whereas the rest is transmitted. Most human tissues—except for fat, air, and bone—have acoustic properties similar to those of water, making them optimal for transmitting ultrasonic energy from the transducer into the body. Moreover, as the wave propagates through a tissue layer, pressure fluctuations induce microscopic shear movements, generating heat through friction and producing thermal and mechanical effects that modify the targeted tissue [10,11].

Focusing is the convergence of sound beams at a specific point, known as the focal zone, achieved through lenses that modulate sound waves to converge at that point through three-phase compensation mechanisms: (1) using a uniform material over an arc-shaped surface, (2) applying diffractive structures, phased arrays or cross-sectional lenses to design a wavefront (zone where the sonic waves converge), and (3) modifying this wavefront through artificial structures to focus on the desired depth and location. In HIFU systems, curved heads are often used to enhance targeting by further increasing wave intensity [12,13].

### 3.2. Transducer Design and Principle of Constructive Effect

HIFU equipment consists of an ultrasonic transducer, which generates and receives ultrasonic vibrations. It comprises a piezoelectric crystal that converts electrical energy into mechanical energy and vice versa, a dense, highly attenuating support structure that controls crystal vibration and resolution, and an anti-wear plate. The most common type is the concave focusing transducer with fixed opening and focal length, which helps to determine the focal point owing to its geometry and mechanical movement. In turn, the electronic steering of the ultrasonic beam provided by this transducer helps achieve more precise control of the focal point location, enabling specific tissue to be reached [14,15].

The waves emitted by the transducer can interfere with one another, leading to either amplification or attenuation of wave amplitude. This phenomenon, known as interference, is classified as destructive when amplitude decreases and constructive when it increases. This feature is important because the ability to pinpoint the location and intensity of the ultrasonic wave within the specific tissue (focal point) is achieved through the principle of constructive interference, which occurs when two or more in-phase waves meet and overlap their peaks and valleys. This results in a wave with a greater amplitude that can penetrate the tissues and perform its therapeutic function [16,17].

### 3.3. Conversion of Electrical Energy into Mechanical Energy

Piezoelectricity, also known as “pressure electricity,” is a property of certain dielectric materials that allows them to become electrically polarised when subjected to mechanical stress, thereby generating an electric charge and producing a piezoelectric effect. Conversely, the application of an electric field can induce mechanical deformation in these materials, producing mechanical waves from the electric charge, a phenomenon known as the inverse piezoelectric effect [18,19].

In HIFU systems, transducers contain piezoelectric materials, which may be lead-free crystals (e.g., sodium bismuth titanate) or lead-based crystals (e.g., lead zirconate titanate). When an electrical pulse is applied to these crystals, their internal dipole structure realigns. This realignment causes their contraction and expansion, producing vibrations in the crystal that eventually generate waves at certain frequencies. Thus, the electric fields produced convert electrical or kinetic energy into mechanical energy via the inverse piezoelectric effect [14,20].

### 3.4. Penetration Frequencies and Depths

Sound frequencies are defined as the number of times a sound is repeated, produced by the organised movement of molecules and atoms that cause the periodic compression of matter, creating waves. Depending on frequency, waves may be audible to humans (16 Hz–20 kHz) or ultrasonic (>20 kHz). In HIFU, waves in the 0.8–20 MHz range are used, with the frequency varying depending on the tissue being treated. This wave is inversely proportional to penetration, as higher-frequency waves have shorter wavelengths and are absorbed more quickly by adjacent tissue than lower-frequency waves [21,22].

Depth of penetration is not determined by frequency alone, but by the combined effect of frequency, transducer geometry, and beam focusing. As illustrated by the devices summarised in Table 1, commercially available transducers combining a frequency of approximately 4 MHz typically focus at a depth of ~4.5 mm, reaching the SMAS, whereas transducers operating at 7 MHz are commonly focused more superficially, at depths of approximately 1.5–3.0 mm, targeting the deep or superficial dermis. Very high frequencies (up to 20 MHz) have been described in experimental cutaneous ultrasound systems (67) but are not represented among the clinically available platforms discussed in this review [23,24].

## 4. Limitations of Conventional HIFU Technologies and the Potential Advance of Multi-Transducer HIFU Platform

Despite the established efficacy of HIFU, first- and second-generation devices had certain limitations. These included patient discomfort or pain during treatment, uneven thermal energy distribution due to static application, prolonged procedure times for full-face treatments, and challenges in effectively treating anatomically complex or highly contoured regions. Furthermore, although single-depth targeting is effective for deep structural support, comprehensive rejuvenation frequently requires addressing multiple skin layers, from the dermis to deeper facial planes, to optimise both tightening and textural outcomes [25].

For this reason, studies are currently being conducted using line-type and pen-type applicators on this platform, which could represent a technological evolution specifically developed to overcome some of these challenges. Its multi-transducer architecture delivers energy at various depths, ranging from 1.5 mm to 13 mm. Therefore, in theory, a more complete, three-dimensional tissue remodelling could be achieved within a single session. Additionally, this technique includes a pen-type applicator, which shifts the kinetics from static to dynamic, potentially offering advantages over conventional HIFU devices [26].

However, it is important to note that, unlike microfocused ultrasound with visualisation (MFU-V; Ultherapy), most alternative HIFU platforms, including Liftera, lack integrated real-time ultrasound imaging. This absence may increase the risk of imprecise targeting and collateral tissue injury, underscoring the need for operator expertise and careful patient selection. Independent comparative clinical trials directly evaluating HIFU against platforms with visualisation remain limited. In contrast, these features suggest theoretical advantages in ergonomics and three-dimensional remodelling; robust histological and clinical correlation studies are still required to confirm superiority or equivalence to established systems.

### Mechanism of Action of Liftera

The system is available in two primary applicator types: Liftera-A (which uses line cartridges for a stamping-type application over wider areas) and Liftera-V (which employs a pen-type applicator with single transducer cartridges for dynamic “painting” or moving techniques in more contoured or smaller areas); It incorporates multi-depth cartridges designed to target different tissue planes, including the superficial dermis, deep dermis, and SMAS. Although these transducers operate at approximately 4 MHz and 7 MHz, focal depth should not be understood as determined solely by frequency. In focused ultrasound systems, treatment depth depends on the combined effect of frequency, transducer geometry, focal configuration, cartridge design, and other device-specific parameters [26].

Thus, frequency influences attenuation and tissue interaction, but it does not intrinsically or automatically assign a fixed penetration depth such as 1.5, 3.0, or 4.5 mm. This multi-layered approach allows the operator to create a homogeneous, three-dimensional distribution of ultrasonic waves, theoretically ensuring that energy is delivered precisely to the targeted tissue compartments while minimising collateral damage to non-targeted structures [26].

Line-type and pen-type applicators of this platform employ convergent ultrasound waves that increase focal temperatures to 60–70 °C, inducing immediate collagen contraction and fibroblast activation, thereby stimulating neocollagenesis of collagen types I and III. Owing to its line and pen applicators, the system addresses multiple layers from the superficial dermis to the SMAS, promoting gradual, sustained remodelling of the extracellular matrix (ECM) with long-term lifting and rejuvenating effects. Its control unit adjusts energy, shot density, and focal depth in real time based on each patient’s anatomy, optimising safety and reproducibility. The pen applicator further enhances comfort by delivering energy continuously and homogeneously, reducing the perception of individual pulses and significantly improving comfort during treatment [26].

## 5. Biological Effects of HIFU

Focused HIFU beams can produce a cascade of thermal or mechanical effects on tissues, or a combination of both, depending on the applied parameters, ultimately leading to tissue destruction or remodelling. It is important to note that aesthetic HIFU/MFU-V devices are deliberately engineered to operate below the inertial-cavitation threshold and to elicit predominantly thermal effects, producing discrete, controlled coagulation zones rather than generalised mechanical tissue destruction; the mechanical and cavitational phenomena described below are presented for physical completeness and because they are relevant to other, non-aesthetic HIFU applications, not because they represent the intended mechanism of cosmetic skin-tightening procedures [27].

The core operating principle of this technology is the conversion of acoustic (mechanical) energy into thermal energy, with the aim of eliciting biological effects in the deep dermis, namely the SMAS and adipose tissue. Thermal coagulation–the predominant mechanism deliberately exploited by aesthetic HIFU-MFU-V devices– is achieved by absorbing ultrasonic waves that induce vibration in the molecules in the region where the ultrasound beam is focused. Within these discrete, spatially confined thermal coagulation points, friction rapidly increases the temperature of the target tissue to approximately 60–85 °C in a few seconds. This results in irreversible cellular injury, including protein denaturation and coagulation, with rapid focal coagulative necrosis [28,29].

Multiple studies have demonstrated the high precision of HIFU, which produces small, controlled thermal effects that minimise damage to adjacent structures, thereby establishing HIFU as an effective technique for aesthetic applications [16]. In addition to its thermal effects, focused ultrasound waves can also induce mechanical effects, such as radiation force and acoustic cavitation [30]. The latter is known to cause irreversible injury by mechanically altering cell membrane permeability and damaging cell structure [29]. Acoustic cavitation is a phenomenon that occurs at high acoustic intensities, during which gas microbubbles are created and oscillate within the tissue, interacting with the ultrasound field.

Two types of acoustic cavitation have been studied: stable and inertial cavitation. Stable cavitation occurs when bubbles oscillate with a stable size and constant shape, generating localised shear forces that cause a slight increase in temperature. However, above a certain pressure threshold, the bubbles collapse energetically, resulting in increased temperature and a strong pressure wave that causes collateral damage to surrounding healthy tissues, a phenomenon known as inertial cavitation [31].

It is noteworthy that HIFU can be used to generate non-thermal effects for tissue destruction, known as histotripsy. In this technique, repeated short-duration pulses (from micro- to milliseconds) with low duty cycles induce rapid bubble oscillation, mechanically disrupting soft tissue without significant temperature elevation [32].

A second mechanical effect is radiation force, the momentum transferred from the propagating wave to the absorbing tissue, which produces displacement and deformation [31]. In the acoustic power and pulse regimes used in conventional aesthetic HIFU/MFU-V protocols, however, exposures are kept below the inertial cavitation and histotripsy thresholds, so these phenomena do not constitute the intended mechanism of action. Rather than amplifying tissue destruction, radiation force at intensities below the cavitation threshold primarily drives tissue displacement and improved energy coupling. At the same time, the therapeutic outcomes sought (collagen remodelling and skin tightening) continue to depend on the discrete, controlled thermal coagulation points described above.

In parallel with these controlled thermal coagulation effects, HIFU also stimulates gradual tissue repair. Following focal cell necrosis within the coagulation point, a tissue remodelling response is triggered to restore structural integrity at the treated site [33]. As mentioned above, the rapid focal temperature increase induces collagen fibre denaturation and contraction, eliciting an acute but controlled inflammatory response.

This local thermal injury strongly stimulates fibroblast activation, migration, and proliferation. As a result, a wound-healing response is triggered, leading to increased synthesis of new collagen types, initially collagen type III, which is subsequently replaced by collagen type I, elastin fibres, and other ECM components. This response reduces overall skin laxity, making it more resistant and elastic. Thicker, shorter collagen fibres result from the breaking of intramolecular hydrogen bonds, which forces the collagen chains to become more stable [30,34], Figure 1.

HIFU achieves skin rejuvenation through four synergistic biological mechanisms. The thermal effects generate precise focal heating between 60 and 85 °C, creating controlled microthermal injury zones measuring approximately 1 × 10 mm. Simultaneously, mechanical effects primarily contribute through subinertial radiation force, which aids tissue displacement and energy coupling. The rapid focal temperature increase induces collagen fibre denaturation and contraction, eliciting an acute but controlled inflammatory response. This local thermal injury strongly stimulates fibroblast activation, migration, and proliferation, increasing neocollagenesis and elastogenesis. At the molecular level, HIFU modulates key pathways by reducing Caveolin-1 expression, activating ERK1/2 signaling, and inhibiting STAT3, thereby promoting fibroblast proliferation and sustained collagen production. These integrated processes lead to progressive tissue remodeling, with clinical improvements in skin tightness and texture becoming most apparent 2–3 months after treatment as new collagen matrix formation reaches its peak. The treatment’s effectiveness stems from its ability to selectively target deeper skin layers, such as the SMAS, while sparing the epidermis, offering a non-invasive approach to aesthetic enhancement with results lasting up to 18 months.

Preclinical studies have further elucidated these mechanisms. For example, Oh et al. demonstrated that HIFU promotes dermal regeneration by increasing collagen and elastin fibre density through modulation of caveolin-1 (Cav-1), a protein involved in cellular senescence and collagen synthesis. A reduction in Cav-1 levels was observed after HIFU administration, which, in turn, led to enhanced ERK1/2 phosphorylation and decreased p53 activation, a protein involved in cell cycle regulation. Similarly, reductions in p21 levels and increases in cyclin levels and other markers of cell proliferation were observed, which favour the synthesis of collagen and elastin in the skin [35].

Owing to its multi-depth targeting, line-type and pen-type applicators of this platform could induce a more generalised and stratified fibroblast response, leading to a more three-dimensionally integrated neocollagenesis and a significant improvement in overall skin architecture compared to single-depth treatments. In addition to the production of new collagen, HIFU-induced thermal damage promotes the release of several growth factors, particularly transforming growth factor-beta (TGF-β), which plays a key role in ECM remodelling by stimulating fibroblast proliferation, differentiation into myofibroblasts, and increased collagen deposition [36,37,38].

This damage also leads to a transient, regulated increase in matrix metalloproteinases (MMPs), a group of endopeptidases that degrade denatured collagen fibres and other damaged ECM components. This process is carefully balanced by the simultaneous increase in tissue inhibitors of metalloproteinases (TIMPs), preventing excessive degradative action [39]. This orchestrated balance between MMPs and TIMPs is essential for the efficient removal of damaged matrix components while allowing the organised deposition and maturation of newly synthesised collagen and elastin.

Furthermore, HIFU-induced hyperthermia upregulates heat shock proteins (HSPs), particularly HSP47, which functions as a molecular chaperone to facilitate the correct folding of the collagen triple helix, prevent aggregation, and ensure structural integrity, contributing to the durability of the newly formed tissue [40,41,42], Figure 2.

HIFU triggers a cascade of molecular events in dermal fibroblasts, leading to skin rejuvenation. HIFU downregulates Caveolin-1 (Cav-1), activating phosphorylated ERK1/2 and suppressing p53/p21 to enhance fibroblast proliferation. Concurrently, HSP47 upregulation inhibits STAT3, thereby relieving its repression of collagen synthesis. Mechanical stress from HIFU stimulates TGF-β, further promoting extracellular matrix remodeling through collagen I/III and elastin production. These synergistic pathways, mediated by thermal and mechanical effects, underlie HIFU’s clinical efficacy in improving skin laxity and texture, with peak collagen regeneration occurring 2–3 months post-treatment.

The overall effect of these complex molecular processes is a comprehensive remodelling of the dermal architecture, accompanied by increased skin firmness and texture, reduced wrinkle depth, and restored viscoelastic properties, all hallmarks of effective aesthetic rejuvenation.

## 6. Clinical Applications of HIFU

The unique capabilities of HIFU have made it a valuable tool in multiple medical and aesthetic applications, particularly in facial rejuvenation and body contouring. One of its main applications in aesthetic dermatology is facial and neck rejuvenation. This technique can be used to treat the underlying skin layers, including SMAS, deep dermis, and subcutaneous fat, to improve skin tightening and elasticity, reduce the appearance of wrinkles, and enhance facial contour. Furthermore, due to its precise targeting and controlled thermal effects, HIFU allows selective treatment of specific areas, such as the eyebrows, cheeks, jaw, and neck, without damaging surrounding healthy tissue or compromising the epidermis [43].

HIFU transducers generate ultrasound waves that are directed to different depths within the skin. At 4.5 mm, waves reach SMAS, inducing denaturation and contraction of the existing collagen, resulting in an immediate tightening or lifting effect [44]. Subsequently, mediators such as interleukins (IL-1 and IL-6), tumour necrosis factor (TNF-α), and other factors (FGF-2, IGF-1, TGF-β, and VEGF) stimulate fibroblast synthesis of new collagen and elastin [45]. Therefore, denatured collagen fibres are gradually replaced by newly formed fibres, resulting in thicker, firmer skin with reduced wrinkles and fine lines.

Although some people notice improvements in skin firmness and texture immediately after treatment, the most significant effects (collagen production and skin rejuvenation) require time to develop. Therefore, the most noticeable clinical results are observed within 2–3 months after treatment. The benefits of HIFU treatment typically last up to 18 months, depending on individual skin characteristics and lifestyle factors [30].

Studies suggest that the increased temperature generated by HIFU can induce the expression of a heat shock protein (HSP47), related to fibroblast proliferation and collagen production through the blockade of the STAT3 signalling pathway. HSP47 expression increases 3 months after the procedure, which may explain the continued skin tightening observed until at least three months after the treatment [46]. Beyond facial rejuvenation, HIFU has also been investigated for body contouring applications, including the reduction of localised fat deposits and subcutaneous adipose tissue (SAT).

HIFU-induced thermal and mechanical effects can be harnessed to induce controlled damage to fat cells, leading to their progressive elimination and, in turn, a reduction in unwanted fat deposits [47]. The reduction in SAT thickness is achieved through heat-induced coagulative necrosis of adipocytes, which occurs at temperatures above 60 °C. However, hyperthermia in the range of 40–45 °C can also trigger adipocyte apoptosis. Notably, a study in animals fed a high-fat diet demonstrated that HIFU, in response to increased temperature, induces both nuclear and mitochondrial p53 expression, along with downregulation of BCL2/BCL-xL (anti-apoptotic signalling) and upregulation of BAX/BAK (pro-apoptotic signalling). These molecular changes lead to apoptosis and autophagy, ultimately resulting in reduced SAT thickness [48].

Similarly, mechanical effects alter cell membranes. Ultrasound waves generate pressure cycles that increase and decrease, forming gas bubbles within the tissue. When these bubbles implode, they release energy, causing mechanical disruption to the target adipocytes [49]. The contents of the damaged adipocytes, primarily triglycerides, are dispersed in the interstitial spaces before being transported by the lymphatic system. Histological analyses of adipose tissue treated with HIFU have revealed vacuole formation and pyknotic nuclei within the extracellular space. These cellular remnants and lipids are subsequently cleared by macrophages [48]. Debris removal and tissue repair typically require 8 to 12 weeks, resulting in a gradual reduction in fat-layer thickness [33].

Moreover, the multi-transducer architecture is expected to deliver results comparable to, or even superior to, those of other HIFU systems due to its pen-like applicator, which has shown improved performance in anatomically complex areas, such as the periorbital region and chin, while also enhancing patient comfort [5].

## 7. Considerations and Safety Profile

Achieving optimal outcomes in non-invasive aesthetic treatments, such as HIFU and new line-type and pen-type applicators of this platform, which use advanced focused ultrasound technologies, largely depends on appropriate patient selection and rigorous pre-procedure management. Ideal candidates are typically individuals aged 30 to 65 with mild-to-moderate skin laxity in areas such as the face, neck, or body. Clinical signs may include submental laxity, loss of jaw definition, brow ptosis, fine lines, or wrinkles, all of which are associated with good skin quality and realistic expectations regarding the results of a non-surgical procedure. Adequate dermal thickness favours a more favourable response, whereas thin, atrophic, or severely photodamaged skin may yield limited outcomes or carry a greater risk of complications [34,50].

Although the benefits of HIFU are well documented, its use is not risk-free. Most reported adverse events are mild, transient, and self-limiting. The most common adverse events are dermatological (specifically localised erythema and oedema), which usually resolve within 24–72 h. Pain during the procedure, often described as burning or stinging, is attributed to activation of dermal nociceptors by the delivered energy [30,51].

However, less frequent but clinically relevant events have been described, such as dysesthesia, contact dermatitis, ecchymosis, and the appearance of persistent linear striae. These have been generally associated with inadequate technical management, such as multiple applications in the same region or insufficient recovery times between sessions. Tissue overheating, a consequence of repeated and inappropriate exposure to thermal energy, can cause subdermal structural damage, fibrosis, localised fat atrophy, and permanent facial asymmetries, negatively affecting aesthetic outcomes and patient satisfaction [52].

From a neurological perspective, the anatomical proximity of the SMAS to the facial nerve’s superficial branches poses a significant risk. The most common manifestations include paresthesia, tingling, numbness, and, to a lesser extent, transient muscle weakness. These symptoms usually appear 3–7 days after the procedure and resolve spontaneously within weeks or months. Their pathophysiology is related to direct thermal damage or neuritic inflammation. Appropriate transducer selection, correct depth targeting, and real-time anatomical visualisation are key to minimising these risks [53,54,55].

Treatment of periorbital regions poses additional challenges due to ocular sensitivity. Incorrect application may cause mild symptoms, such as transient blurred vision, tearing, or conjunctival hyperemia, but severe complications including corneal erosions, stromal oedema, increased intraocular pressure, induced myopia, or traumatic cataracts have also been reported. To mitigate these risks, shots over the supraorbital ridge should be avoided, and protective ocular shields are recommended when treating areas close to the eyes [56].

Although rare, vascular complications may also occur with inappropriate ultrasound use. Thermal injury with endothelial remodelling and fibrosis of the internal carotid artery has been reported, leading to significant stenosis and neurological symptoms in a patient treated in the cervical region. Such findings highlight the importance of using devices with integrated imaging (MFU-V) to avoid applying energy to important vascular structures, particularly in patients with little adipose panniculus [3,57].

Contraindications include active infections or inflammations (herpes simplex, cellulitis), open wounds, severe cystic acne, metallic or electronic devices implanted in the area, pregnancy, and breastfeeding. There are relative contraindications that require caution, such as a history of pathological scarring, autoimmune diseases with skin involvement, bleeding disorders or anticoagulation, recent aesthetic treatments, and focal neurological disorders. Rigorous clinical evaluation includes a thorough history, an objective assessment of skin laxity using validated scales, a detailed discussion of expectations and risks, and informed consent, along with photographic documentation [3].

Pre-procedural management should be individualised according to the patient’s medical history, current medications, bleeding risk and the recommendations of the prescribing physician; in patients receiving anticoagulant or anti-inflammatory therapy, the potential benefit of temporarily withholding these agents should be weighed against the risks of interrupting treatment, rather than applied as a routine requirement. Additional measures include administering prophylactic analgesia or topical anaesthesia to improve tolerance and meticulously preparing the area to be treated. In complex cases or sensitive patients, anxiolytics or nerve blocks may be used. Correct patient selection, together with the operator’s experience and the appropriate use of technology with real-time visualisation, are fundamental pillars for maximising benefits and minimising complications in treatment devices based on HIFU and MFU-V [58].

## 8. Clinical Evidence

The increasing use of HIFU in non-invasive aesthetic procedures has prompted numerous clinical studies to assess its efficacy, safety, and tolerability. Collectively, this body of evidence supports its application in facial and cervical rejuvenation as well as body contouring, documenting significant improvements in skin laxity, contour definition, and reduction of localised fat deposits. However, the dynamic and stamping HIFU platform has few studies. For this reason, none of the studies mentioned use this tool [6,30].

A prospective study by Azuelos et al. in a European population included 20 healthy volunteers who underwent HIFU treatment to improve neck skin laxity and reduce submental fat. Objective and subjective evaluation conducted by evaluators using standardised scales (FACE-OAS and IGIAS) demonstrated significant improvements in the definition of the cervicomental angle and skin laxity, with no major complications reported. The procedure was well tolerated, with mean pain scores varying according to the transducer used for Ultherapy: 5.6 for the D4 (4.5 mm), 4.2 for the M7 (3.0 mm), and 2.05 for the S7 (1.5 mm) [59].

Similarly, a retrospective study by Asiran et al. involving 75 patients, mostly women with Fitzpatrick phototypes II–IV, evaluated HIFU outcomes on the face and neck using 3 mm and 4.5 mm transducers. At 3 months, independent physicians reported clinical improvement rates exceeding 80% in the nasolabial folds, jawline, submental area, and neck. Patient satisfaction exceeded 78% in all treated regions. Adverse events were mild and transient, with pain during the procedure being the most common (25.3%), followed by transient erythema (6.7%), and an isolated case of mandibular paresthesia that resolved spontaneously within 10 days [60].

At the level of systematic evidence, a meta-analysis by Ayatollahi et al., which included 17 studies with a total of 477 participants, found moderate clinical improvement in facial and cervical rejuvenation, both in objective assessment (mean score 2.74/5) and subjective satisfaction (2.68/5). Perceived pain was rated as moderate (4.2/10), with no reports of hyperpigmentation. Although most studies had short follow-up periods and methodological heterogeneity, the findings support HIFU as a safe and effective technique for improving skin laxity and visible signs of ageing [61].

Beyond the exclusive use of HIFU, therapeutic combinations have been explored to enhance its rejuvenating effects. One recent study assessed the combination of microfocused ultrasound, botulinum toxin type A (incobotulinumtoxinA), and hyaluronic acid for neck treatment. Although patient satisfaction reached 100%, only 60% of cases were rated by physicians as clinically improved, whereas 20% showed no change, and another 20% worsened. This finding suggests that although combinations may raise subjective perceptions of improvement, objective results do not always correspond to those expectations [62].

Similarly, a retrospective study examined the effects of microfocused ultrasound with visualisation (MFU-V) in combination with 1:1-diluted calcium hydroxyapatite (CaHA) on the neck and décolleté. At 90 days after treatment, a significant reduction in the severity of cervical and décolleté wrinkles was observed, with mean scores decreasing from moderate to mild. The procedures were well-tolerated, with high patient satisfaction reported [63].

In contrast, multiple studies using HIFU alone have demonstrated consistent objective clinical improvement rates, ranging from 58% to 91%, and patient satisfaction levels of 60% to 90% [64,65]. A recent systematic review analysing 45 clinical and cohort studies published between 2010 and 2024 confirmed the efficacy of HIFU for skin tightening in the lower face, neck, and periorbital region, with a reduction in skin laxity of 18%–30%. In body contouring, circumference reductions of 2.5–4.5 cm were reported, particularly in the abdomen and thighs. Fewer than 5% of patients experienced mild, transient adverse events, such as erythema or oedema, underscoring its favourable safety profile [6].

Unlike other non-invasive technologies, such as radiofrequency, lasers, or combination treatments, HIFU uniquely reaches deep anatomical structures, including the SMAS and platysma muscles, key targets for effective lifting. These findings reinforce the use of HIFU as a robust, safe, and versatile non-invasive alternative, particularly useful for patients seeking natural results with minimal recovery time [57].

However, it is critical to note that the majority of high-quality, independent evidence derives from microfocused ultrasound with visualisation (MFU-V) platforms such as Ultherapy. Studies specifically evaluating the latest HIFU are few and often rely on smaller cohorts or manufacturer-supported data. Direct head-to-head comparisons between HIFU and visualisation-guided systems are currently lacking. While the multi-depth and dynamic applicator features show theoretical promise for simultaneous superficial and deep remodelling with improved comfort, objective superiority has not yet been demonstrated in large independent randomised trials.

In this context, Lio et al. conducted a retrospective study involving 12 participants with Fitzpatrick skin phototypes III–V. They evaluated the efficacy of a single facial HIFU session using multi-depth Ultraformer transducers (2, 4.5, and 6 mm) applied to the forehead, temporal regions, and zygomatic arches. The protocol included 240 precisely distributed thermal coagulation lines, resulting in a significant increase in brow elevation angles (*p* = 0.0003) and objective improvements in periorbital linear measurements at 60 and 90 days. Standardised photographic analysis confirmed a visible lifting effect in the frontal and periorbital region. The procedure was well-tolerated, and no relevant adverse events were reported. This study supports the efficacy of HIFU as a single treatment for upper- and mid-facial rejuvenation, highlighting the clinical value of using transducers of varying depths to address multiple anatomical layers [7] simultaneously.

Undoubtedly, the available evidence supports the use of HIFU as an effective, safe, and well-tolerated modality for facial and neck rejuvenation, with proven clinical benefits in both improved skin laxity and patient satisfaction. Although combined therapies may provide complementary results in selected cases, HIFU alone has proven sufficient to achieve meaningful improvements across diverse patient populations. Nonetheless, variability in treatment protocols, differences in device specifications, and individual patient characteristics highlight the need for careful clinical evaluation. Proper candidate selection, personalised treatment planning, and precise technical execution remain key to optimising outcomes and minimising risks in aesthetic HIFU practice [24,66].

It is important to note that, to date, only isolated studies have included patients with Fitzpatrick skin phototype V within predominantly lighter-skinned cohorts (e.g., Lio et al., whose sample spanned phototypes III–V), and no studies have specifically evaluated the efficacy or safety of HIFU in phototype VI. This scarcity of data in darker skin phototypes represents an important limitation for the generalizability of the evidence. It would be of great interest to elucidate whether HIFU treatment can be safely and effectively applied to all skin tones, and whether it is possible to reach the subcutaneous layers, SMAS, and muscle without inducing injury to the epidermis or dermis. Such studies will be critical to ensuring equitable and safe access to non-invasive aesthetic treatments such as HIFU.

## 9. Critical Assessment and Main Knowledge Gaps Identified for HIFU

One of the main current limitations in the available evidence on line-type and pen-type applicators of this platform is the scarcity of randomised, independent, multicentre clinical trials that rigorously evaluate its clinical performance. To consolidate its position within the therapeutic arsenal of aesthetic dermatology and to enable the formulation of evidence-based recommendations, future research should address several relevant gaps.

First, direct comparative studies are required to evaluate (using both the Type A and Type V applicators) its relative efficacy, safety profile, and patient-reported outcomes against other leading HIFU platforms, such as Ultherapy, Ultraformer, or Sofwave, across different clinical settings. It is also necessary to establish optimal treatment parameters, including energy levels, shot density, number of applications, depth and frequency combinations, and adjustments for variables such as skin phototype (based on the Fitzpatrick scale), anatomical region treated, and degree of tissue laxity [67].

Another research priority is evaluating the long-term durability of results. Although the short- and medium-term effects of HIFU treatments are generally favourable, it is essential to conduct prolonged follow-up studies (≥12–24 months) specifically focused on assessing the persistence of clinical effects and the eventual need for maintenance sessions. In addition, applicator technology deserves further exploration. Objective studies should quantify the possible advantages of the mobile pen-type applicator, both in terms of clinical efficacy (e.g., through 3D imaging or elastography), and in aspects such as treatment time, patient comfort (using validated pain scales), and ergonomics for the operator, compared to static-type applicators [68,69].

In this regard, in vivo human studies are needed to correlate HIFU treatment parameters with specific histological changes, such as the depth and volume of thermal injury zones (TIZs), the amount of new collagen and elastin fibres generated, as well as expression of molecular markers such as TGF-β, HSPs, and the balance between MMPs and their inhibitors (TIMPs) over time. Finally, standardisation of outcome measures should be a methodological priority. Future studies should incorporate objective and validated tools, such as three-dimensional imaging systems (VECTRA^®^, Canfield Antera 3D™), cutometry, and elastography, along with standardised scales for subjective assessment by patients and physicians, such as the Global Aesthetic Improvement Scale (GAIS) or the FACE-Q questionnaire [25].

Although the safety profiles of both HIFU and the newest devices are generally favourable, with most adverse events mild and transient, continuous monitoring and systematic adverse event reporting in well-structured clinical studies remain essential. It is particularly important to generate data in higher Fitzpatrick skin phototypes (V–VI), which are common in Caribbean and Latin American populations, to ensure equitable access and safety. Ultimately, addressing these knowledge gaps through targeted research will be critical for the dynamic and stamping HIFU system to evolve from a promising emerging technology into a validated, evidence-based tool in aesthetic dermatology.

## 10. Conclusions

HIFU has been established as an effective, safe, and minimally invasive modality for treating skin laxity, wrinkles, and facial contouring, as it induces deep tissue remodelling without damaging the epidermis. Its application in aesthetic medicine has demonstrated clinically significant, long-lasting outcomes and high patient satisfaction in mild-to-moderate cases. Nevertheless, this technology continues to face significant challenges, including the need for standardised treatment protocols and individualised therapeutic approaches, as well as the underrepresentation of patients with higher Fitzpatrick phototypes in clinical research.

In this context, line-type and pen-type applicators constitute a technically interesting platform that incorporates multi-transducer technology and dynamic applicators, enabling homogeneous energy distribution across multiple tissue layers and potentially improving patient comfort. Its early safety profile is encouraging, but these potential advantages remain to be confirmed, as they currently rest on limited, non-comparative evidence; independent, randomised, multicenter clinical trials directly comparing HIFU with established platforms with real-time visualisation are needed before its specific efficacy and relative benefits can be considered established.

Looking ahead, integrating technologies such as artificial intelligence for personalised treatment planning and predictive response biomarkers may further enhance the clinical impact of these platforms. HIFU is already a well-established, evidence-based modality in aesthetic dermatology, whereas a multi-depth cartridge HIFU platform should, at this stage, be regarded as a promising emerging platform rather than an equally validated one; its role in next-generation, non-invasive aesthetic therapies will depend on whether rigorous, independent, comparative evidence continues to be generated, particularly in diverse skin populations.

## Figures and Tables

**Figure 1:**
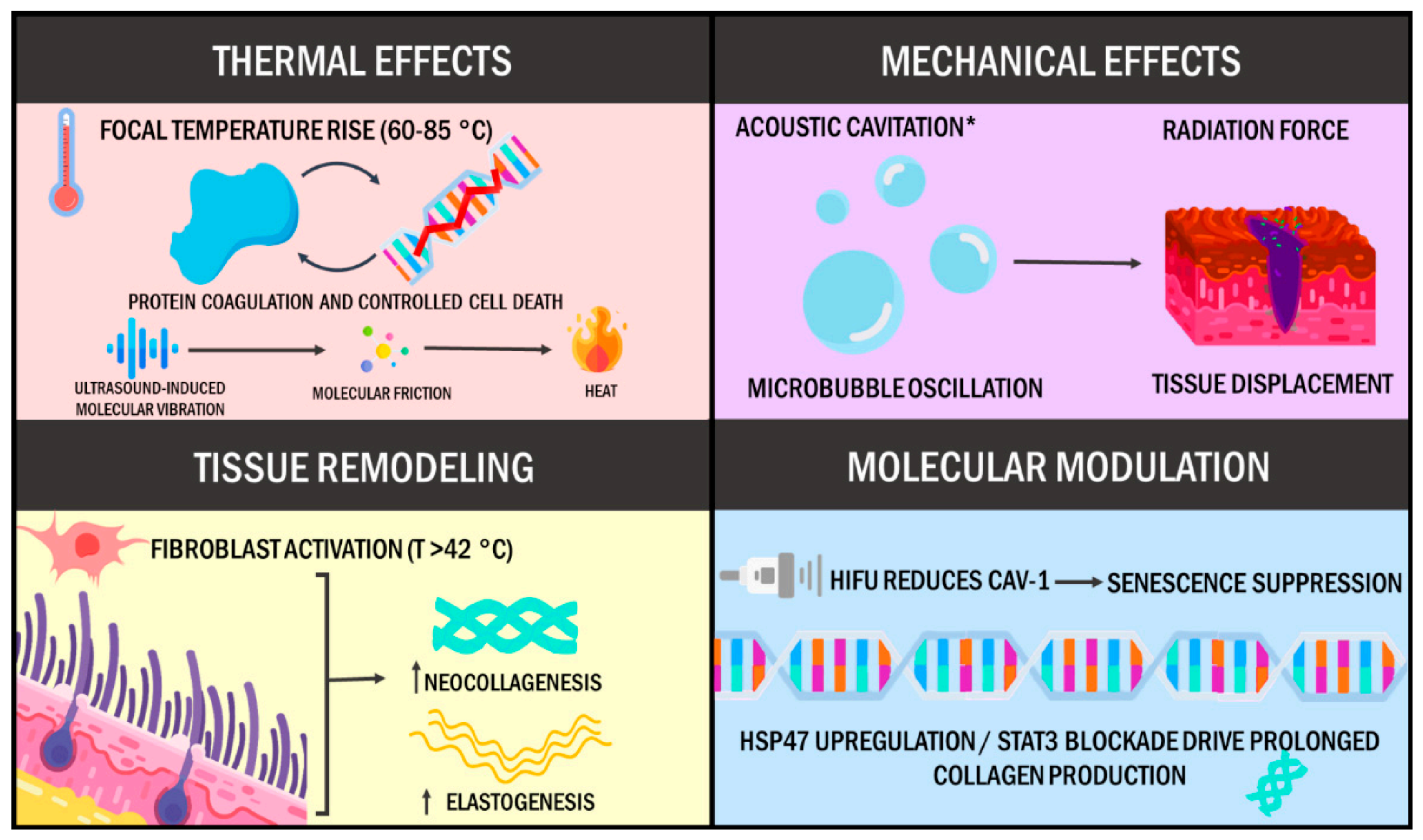
Integrated HIFU biological effects. Abbreviations: HIFU: High-Intensity Focused Ultrasound; CAV-1: Caveolin-1; HSP47: Heat Shock Protein 47; STAT3: Signal Transducer and Activator of Transcription 3; T: Temperature; °C: Degrees Celsius. *: Acoustic cavitation occurs above the intensity thresholds used in aesthetic HIFU/MFU-V protocols; shown for physical completeness and relevance to non-aesthetic applications, not as a routine cosmetic mechanism.

**Figure 2:**
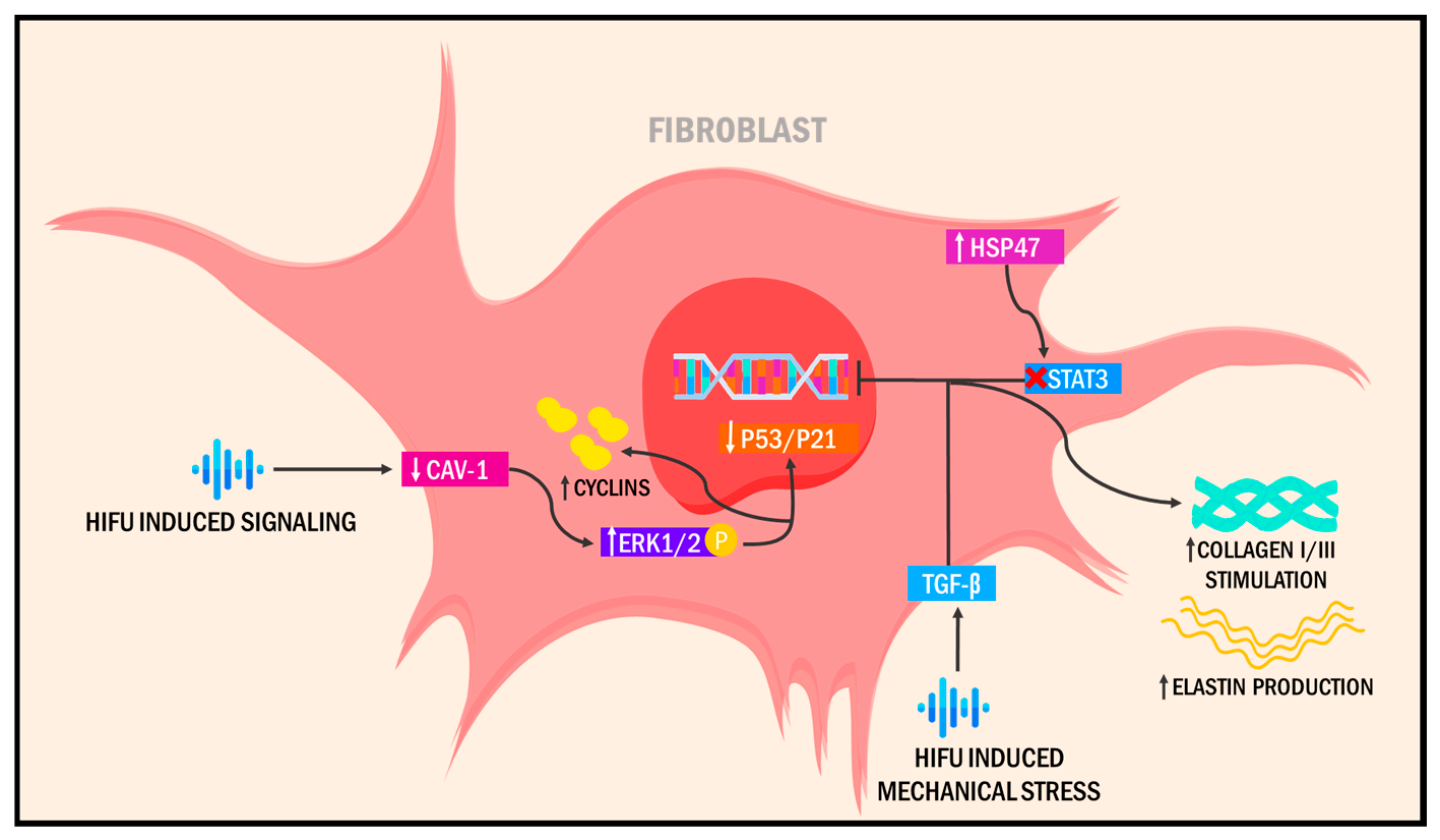
HIFU-Induced molecular signaling cascade in dermal fibroblasts. Abbreviations: HIFU: High-Intensity Focused Ultrasound; CAV-1: Caveolin-1; ERK1/2: Extracellular Signal-Regulated Kinase 1/2; P: Phosphorylation/Phosphorylated; P53: Tumor Protein p53; P21: Cyclin-Dependent Kinase Inhibitor 1A/p21; HSP47: Heat Shock Protein 47; STAT3: Signal Transducer and Activator of Transcription 3; TGF-β: Transforming Growth Factor-beta.

**Table 1:** Clinical evidence supporting the use of HIFU for facial and neck rejuvenation.

Author	Methodology	Clinical Results	Device/Platform	Sample Size	Follow-Up Duration	Outcome Measures	Main Limitations
Azuelos et al.	Prospective study in 20 patients that evaluated the efficacy of HIFU for reducing the double chin and improving neck skin laxity, with outcomes rated by blinded physicians using the FACE-OAS and IGAIS scales.	Clinical improvement was found in cervicomental angle definition and skin laxity. The IGAIS score was 1–5 in 2 patients, 6–10 in 7 patients, and 11–15 in 11 patients. No major complications were reported.	HIFU Doublo-S+ (Hironic) with three transducers: D4 (4.5 mm/4.0 MHz), M7 (3.0 mm/7 MHz) and S7 (1.5 mm/7.0 MHz)	20 patients (13 women, 7 men)	6 months (mean 6.5 months; range 6.11–6.89)	IGAIS score (–5 to 15, 5 blinded evaluators); modified FACE-Objective Assessment Scale for double chin and skin laxity (0–3); SGAIS patient satisfaction.	Assessment based only on subjective scales (no MRI or ultrasound imaging); small sample size; follow-up limited to 6 months; Fitzpatrick skin types V–VI not included
Asiran et al.	Retrospective study in 75 patients with Fitzpatrick skin types II to IV that analysed HIFU outcomes on the face and neck using transducers with 3 mm and 4.5 mm focal depths.	Clinical improvement was greater than 80% in all treated regions; likewise, the satisfaction rate was greater than 78%. Mild adverse effects were reported, such as pain during the procedure (25.3%) and transient erythema (6.7%).	HIFU Doublo-S (Hironic Co.) with two transducers: 4.5 mm/4 MHz (1.2 J) and 3 mm/7 MHz (0.3 J)	75 patients (73 women, 2 men)	3 months (90 days)	4-point improvement scale (0–4) rated by 2 blinded dermatologists across 4 areas (nasolabial, jawline, submental, neck); patient satisfaction (1–4 scale); Krippendorff’s Alpha coefficient	Retrospective design; short follow-up (3 months); lack of objective histologic analysis
Ayatollahi et al.	Meta-analysis pooling 17 studies with a total of 477 patients, published before December 2017, using the Medline, Web of Science, and Scopus databases.	Clinical improvement was moderate for facial and neck rejuvenation, with a score of 2.74/5 on objective assessment and 2.68/5 on subjective satisfaction. Pain was 4.2/10, with no reported hyperpigmentation.	Most studies used Ulthera (USA); 2 studies used Korean devices (UTIMS A1 and Doublo)	477 participants across 17 studies (range 5–52 patients per study)	Variable across studies: 2 to 12 months	Subjective satisfaction, objective improvement, SGAIS and PGAIS (1–5 scales); pain (0–10 scale); edema, erythema and hyperpigmentation	High heterogeneity between studies (I^2^ up to 99%); most were uncontrolled before-after designs (only 1 RCT); limited long-term follow-up (max. 12 months); heterogeneous outcome variables across studies
Doh et al.	Prospective study in 10 patients that evaluated the effect of combined therapy using microfocused ultrasound with visualisation (MFU-V), botulinum toxin type A, and hyaluronic acid for neck rejuvenation.	Patient satisfaction was 100%, and clinical improvement in neck rejuvenation was observed in 60% of the study subjects. It was also noted that 20% of patients showed no improvement and the remaining 20% worsened.	MFU-V (Ultherapy) ± calcium hydroxyapatite (CaHA) (Radiesse), dual depth (7.0 MHz/3.0 mm and 4.0 MHz/4.5 mm), combined with hyaluronic acid filler (Belotero), and incobotulinumtoxin A (Xeomin)	10 Korean patients	3 months	Primary evaluation by 3 blinded evaluators (selection of post-treatment image); secondary scoring table (0–3 points across 4 categories: skin laxity, submental fat, platysmal bands, horizontal neck lines)	Small sample size; short follow-up (3 months); proprietary scoring system requiring further validation; only mild-to-moderate neck aging cases included
Casabona et al.	Retrospective study in 47 patients examining the effects of microfocused ultrasound with visualisation (MFU-V) combined with 1:1 diluted calcium hydroxylapatite for improving the appearance of neck and décolletage lines and wrinkles.	Clinical improvement was found, with a significant reduction in the severity of neck and décolletage wrinkles, with mean scores moving from moderate to mild. Procedures were well tolerated and high satisfaction levels were reported.	MFU-V (Ultherapy) with 7 and 10 MHz transducers + CaHA (Radiesse) diluted 1:1 with lidocaine	47 patients (44 women, 3 men)	90 days (3 months)	Merz Aesthetics, Fabi-Bolton, and Allergan (neck lines) scales; patient satisfaction questionnaire (1–5 scale)	Retrospective design; no comparison against single-agent treatment; unknown whether the combination extends the duration of effect beyond 1 year
Haykal et al.	Systematic review of 45 clinical trials and cohort studies published between 2010 and 2024 evaluating wrinkle improvement and body contour reduction with HIFU.	HIFU demonstrated significant efficacy for skin tightening, with laxity improvements of 18–30%. A circumference reduction of 2.5 to 4.5 cm was also observed in the abdomen and thighs. In addition, fewer than 5% of patients experienced mild adverse effects, such as erythema and swelling.	Multiple HIFU/MFU-V platforms depending on the study reviewed (e.g., Ultraformer, Ultherapy, Sofwave)	45 clinical trials and cohort studies included (2010–2024)	Variable across studies; improvements visible from 3 months and sustained up to 1 year	% improvement in skin laxity; body circumference reduction (cm); adverse effect rates; patient satisfaction	Lack of standardised treatment protocols; scarce data on darker skin phototypes; variability in patient selection criteria and device settings across studies; level of evidence 3
Lio et al.	Retrospective study with 12 participants of Fitzpatrick skin types III to V that evaluated the efficacy of a single session of facial HIFU using transducers with multiple penetration depths applied to the forehead, temporal regions, and zygomatic arches.	A significant increase in eyebrow elevation angles (*p* = 0.0003) and objective improvements in periorbital linear measurements was observed, with a clear lifting effect in the anterior and forehead regions. The procedure was also well tolerated, with no relevant adverse effects reported.	ULTRAFORMER III (Classys) with 3 transducers: MF2 (2 mm/5.5 MHz), L4–4.5 (4.5 mm/4 MHz) and W2-6.0 (6 mm/2 MHz)	12 participants (*n* = 24 hemifaces; 2 men, 10 women)	90 days	13 anatomical landmarks: 4 angular and 5 linear assessments (eyebrow elevation, palpebral length, canthus-oral-nasal angle, tragus-oral length, among others)	No control group; no comparison with a single transducer; small sample size; Asian skin only; effect of repeated sessions not determined

Abbreviations: HIFU: High-Intensity Focused Ultrasound; FACE-OAS: FACE-Objective Assessment Scale; IGAIS: Investigator Global Aesthetic Improvement Assessment Scale; CaHA: Calcium Hydroxyapatite; MFU-V: Microfocused Ultrasound with Visualisation; MRI: Magnetic Resonance Imaging; PGAIS: Physician Global Aesthetic Improvement Scale; RCT: Randomized Controlled Trial; SGAIS: Subject Global Aesthetic Improvement Scale; VAS: Visual Analog Scale.

## Data Availability

No new data were created or analyzed in this study. Data sharing is not applicable to this article.
